# Geochemical and Spatial Distribution of Topsoil HMs Coupled with Modeling of Cr Using Chemometrics Intelligent Techniques: Case Study from Dammam Area, Saudi Arabia

**DOI:** 10.3390/molecules27134220

**Published:** 2022-06-30

**Authors:** Mohamed A. Yassin, Bassam Tawabini, Abdulaziz Al-Shaibani, John Adedapo Adetoro, Mohammed Benaafi, Ahmed M. AL-Areeq, A. G. Usman, S. I. Abba

**Affiliations:** 1 Interdisciplinary Research Center for Membrane and Water Security, King Fahd University of Petroleum and Minerals, Dhahran 31261, Saudi Arabia; mohamedgadir@kfupm.edu.sa (M.A.Y.); bassamst@kfupm.edu.sa (B.T.); benaafi@kfupm.edu.sa (M.B.); ahmed.areeq@kfupm.edu.sa (A.M.A.-A.); 2 College of Petroleum Engineering and Geosciences, King Fahad University of Petroleum and Minerals, Dhahran 31261, Saudi Arabia; 3 Ministry of Environment, Water, and Agriculture, Riyadh 11195, Saudi Arabia; shaibani@kfupm.edu.sa; 4 Centre for Environmental Management and Control, Enugu Campus, University of Nigeria, Nsukka 410001, Nigeria; dapoadetoro@yahoo.com; 5 Operational Research Centre in Healthcare, Near East University, TRNC, Mersin 10, Nicosia 99138, Cyprus; abdullahigusman@gmail.com; 6 Department of Analytical Chemistry, Faculty of Pharmacy, Near East University, TRNC, Mersin 10, Nicosia 99138, Cyprus

**Keywords:** artificial intelligence, trace metals, topsoil, spatial distribution, Saudi Arabia

## Abstract

Unconsolidated earthen surface materials can retain heavy metals originating from different sources. These metals are dangerous to humans as well as the immediate environment. This danger leads to the need to assess various geochemical conditions of the materials. In this study, the assessment of topsoil materials’ contamination with heavy metals (HMs) was conducted. The material’s representative spatial samples were taken from various sources: agricultural, industrial, and residential areas. The materials include topsoil, eolian deposits, and other unconsolidated earthen materials. The samples were analyzed using the ICP-OES. The obtained results based on the experimental procedure indicated that the average levels of the heavy metals were: As (1.21 ± 0.69 mg/kg), Ba (110.62 ± 262 mg/kg), Hg (0.08 ± 0.18 mg/kg), Pb (6.34 ± 14.55 mg/kg), Ni (8.95 ± 5.66 mg/kg), V (9.98 ± 6.08 mg/kg), Cd (1.18 ± 4.33 mg/kg), Cr (31.79 ± 37.9 mg/kg), Cu (6.76 ± 12.54 mg/kg), and Zn (23.44 ± 84.43 mg/kg). Subsequently, chemometrics modeling and a prediction of Cr concentration (mg/kg) were performed using three different modeling techniques, including two artificial intelligence (AI) techniques, namely, generalized neural network (GRNN) and Elman neural network (Elm NN) models, as well as a classical multivariate statistical technique (MST). The results indicated that the AI-based models have a superior ability in estimating the Cr concentration (mg/kg) than MST, whereby GRNN can enhance the performance of MST up to 94.6% in the validation step. The concentration levels of most metals were found to be within the acceptable range. The findings indicate that AI-based models are cost-effective and efficient tools for trace metal estimations from soil.

## 1. Introduction

Soil is one of the most important natural resources in the environment. It is continuously evolving by weathering rock materials, the accumulation of decaying plant matter, eolian deposits, fluvial/marine deposits, etc. Soil provides vital living space to humans and substrate for many microbes, plants, and animal species [1]. Therefore, proper monitoring, protection, and preservation methods of soil are among the primary environmental sustainability objectives. Trace metals are considered major pollutants in soil [2]. These materials are found naturally in the environment with various variable concentrations reported by different environmental scientists. Moreover, various artificial sources such as pollution have been reported [3]. The abnormal levels accumulated may directly impact human health and the ecosystem [4]. It is equally important to note that there is a high tendency for heavy metals to accumulate in the food chain, owing to their sophisticated nature. Human-induced metal accumulations come from various sources [5], ranging from vehicular emissions to industrial waste, fossil fuel combustion, and municipal waste residue disposal.

Although authorities require environmental impact assessment and monitoring reporting, only very few environmental soil studies based in Saudi Arabia have been reported. Reported studies were performed in the areas of Jizan City [6], Red Sea coastal line [7], Jeddah City [8], and Wadi Hanifah, Riyadh, Saudi Arabia [9]. Arif and Hashem [6] studied soil samples in five localities in Jizan City for metal contents and fungal flora. They observed that the soil contained high Pb, Cu, Ni, Mn, and Cd concentrations. Al-Hefner et al. [7] measured heavy element concentrations in the surface soil along the Red Sea coastline using the ICP-MS technique. They reported high Sr, Hf, and U concentrations among the 28 elements found in the soil. Kadi [8] investigated soil samples from roads in Jeddah City. Samples were analyzed for nine elements, and only Pb and Zn were found in higher concentrations. They reported that traffic conditions on the roads are the main reason for higher concentrations of these two elements. Al Yemeni et al. [9] studied Wadi Hanifah, which served as a natural wastewater drainage system for the City of Riyadh. They found high levels of Cd, Ni, Pb, and Zn in both the wastewater and soil sediments of the region. Due to these trace elements’ hazardous nature, it is, therefore, necessary for environmental scientists to identify the degree, concentrations, and primary sources of heavy metals from the agricultural, residential, and industrial soils.

The application of modeling techniques such as the artificial intelligence (AI)-based approach provides a cost-effective, powerful, and efficient strategy for estimating and predicting heavy metals, which can be employed to solve various challenges and issues. Using computational methods such as AI can minimize the financial stress related to earth science, environmental engineering, and spatial geology based on time, cost, space, and labor requirements. Computational techniques were developed recently to solve issues and challenges that cannot be solved using different mathematical models, classical approaches, and linear statistical methods [10,11,12,13]. The robust application of AI techniques is not only limited to the mechanisms involved in minimizing or removing heavy metals, but also can be used in system identification in different fields of science and technology [14,15,16,17,18,19,20].

**Figure 1:**
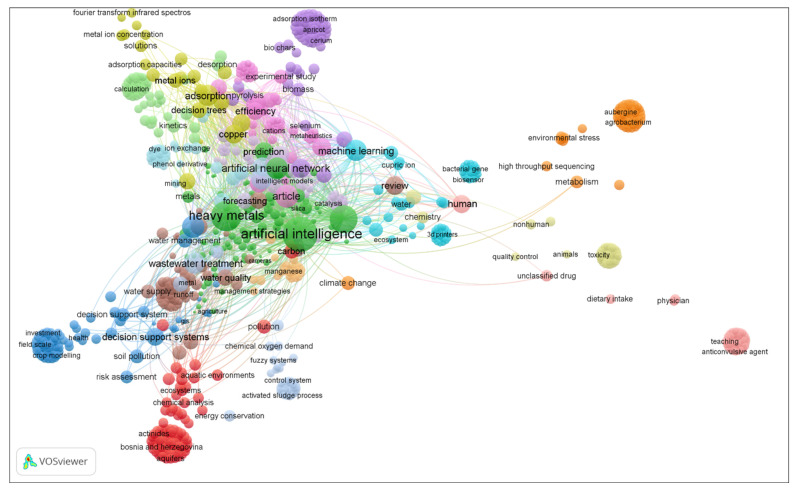
Major keywords used in AI technique analysis on HMs.

The supremacy of computational techniques is related to various factors, such as the development of the models, the kind of learning network to be implemented, and the structure of the network [21,22,23,24]. Even though numerous research works on the implementation of AI-based techniques on heavy metals have been reported in the technical literature, several characteristics could be explored regarding the long-term applications of heavy metal modeling. Hence, to our current knowledge, no study in the technical literature reports the prediction of Cr concentration (mg/kg) using GRNN and Elm NN models in Saudi Arabia. Therefore, this study evaluated the level of heavy metals (HMs)/trace metals in the soil samples taken from different areas of Dammam region, Kingdom of Saudi Arabia. The literature search revealed no reliable reports on the distribution of trace metals in soils of Dammam metropolitan areas, especially using AI-based models. Therefore, this study aims to assess the spatial distribution of heavy metals around the Dammam area, east of Saudi Arabia. Moreover, the study equally employed the application of two AI-based models in the form of generalized neural network (GRNN) and Elman neural network (Elm NN) models, as well as a classical multivariate statistical technique (MST) as a linear method for the prediction of Cr concentration (mg/kg). Another approach to understanding the wide range of applications and connection strength of AI-based models and HMs is by using a bibliographic survey. A survey of the reported literature on the database Scopus yielded the finding that about 200 peer-reviewed papers from between Jan 1985 and June 2022 adopted over the feasibility of a wide interest in HMs and AI-based techniques (see, Figure 1). Figure 1 shows that 1000 keywords occurring between those studies, indicating the deep interest and implementation of this field. In addition, the popularity of this study topic can be investigated in different regions throughout the world, with the bulk of the countries producing the output related to AI and HMs using the same data.

## 2. Material and Methods

### 2.1. Study Location

The city of Dammam is located from latitude 26°20′18″ to 26°32′51″ and longitude 49°49′54″ to 50°09′17″. The city of Dammam is the capital city of the Eastern Province of Saudi Arabia. It is regarded as the largest oil region in the world. It houses the judiciary, administrative, and other government agencies and departments. A large percentage of the province’s population resides in this area. Based on its geographical location, its closeness with the Arab Gulf countries enables it to be a great tourist center. It equally experienced various forms of industrialization and urbanization activities recently.

**Figure 2:**
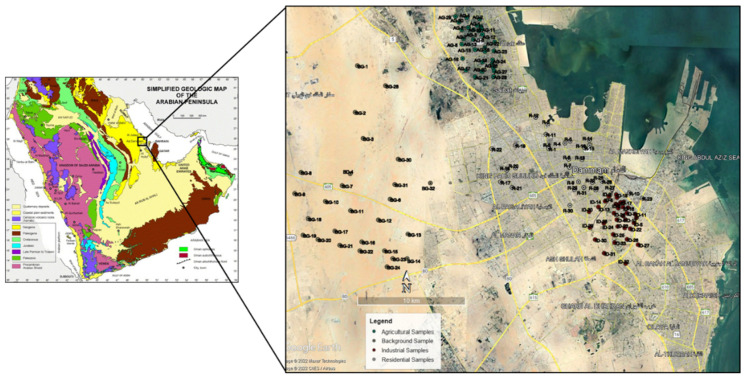
Sample locations used in the current study.

The city of Dammam is also surrounded by many farms that produce dates and other fruits and vegetables. It also has two main industrial cities for small to medium-size industries. Dammam has recently experienced migration, sub-urbanization, and rapid industrialization. A geological map with agricultural (AG), industrial (ID), residential (R), and background (BG) areas was considered and is presented in Figure 2.

The classification of soil types is based on the grain size distribution and soil consistencies. It provides an avenue through which research related to soil can be addressed in a rigorously systematic manner. The unified soil classification system was adopted in classifying the soil. The soil ID is classified as coarse gravel if more than 50% of the soil is gravel, and coarse sand if more than 50% of the soil is sand. The following conditions were also used in classifying the soil: 1 < Cc < 3 and Cu ≥ 4 for gravel; 1 < Cc < 3 and Cu ≥ 6 for sand; P = poorly graded: <5% fine, Cu < 6, and 1 > Cc > 3; W = well-graded: <5% fine, Cu ≥ 6, and 1 ≤ Cc ≤ 3; M = silty: >12% fine; C = clayey: >12% fine.

Soil types in the selected sampling location are as follows:

- For the AG samples, it is mainly poorly graded sand with silt to silty sand;
- For the ID samples, it is mainly silty sand to poorly graded sand with silt and gravel;
- For the R samples, it is mainly well-graded sand with silt and gravel to silty sand;
- For the BG samples, it is mainly poorly graded sand with silt to silty sand.

### 2.2. Sampling Method

For sampling purposes, the Dammam region is in the southern part of Saudi Arabia, whereby the dataset was classified into agricultural, industrial, background, and residential areas. Thirty-three representatives from each of the classes were taken, with a total of one hundred thirty-two samples. The locations of these samples are presented in Figure 2. The procedure involves collecting different soil samples from other locations, recorded by miniaturized and portable positioning system instruments. The samples were then stored using a polythene bag safely in the laboratory. The topsoil samples were collected over a two-month period (i.e., February to March 2014). The procedure is considered as a powerful acid digesting process capable of dissolving all elements that are naturally widespread in the environment.

### 2.3. Geochemical Analysis of Soil Samples

The samples were prepared as per the USEPA method 3050B to digest soil, sediment, and sludge. The samples were then analyzed by inductively coupled plasma-optical emission spectrometer (ICP-OES) (SPECTRO Analytical Instruments GmbH & Co. KG, Kleve, Germany) as per USEPA method 200.7, revision 4.4 [25]. The chemical reagent employed complied with the standards of the ACSCAR (American Chemical Society’s Committee on Analytical Reagents). Distilled water (DI), concentrated nitric acid (HNO_3_), concentrated hydrochloric acid (HCl), and 30% hydrogen peroxide are examples of such reagents (H_2_O_2_). Because the digestion required the use of acid, it was carried out under a fume hood, under the supervision of an expert, and with the certified and recommended laboratory safety equipment. The equipment was calibrated using the multi-elemental standardization approach, which consists of six different working standards incorporated with a single blank used to identify the tools’ accuracy and suitability. Quality control measurements were equally used for each batched processing of the samples under consideration. Each batch was composed of 20 different samples.

### 2.4. Proposed Machine Learning Methodology

**Figure 3:**
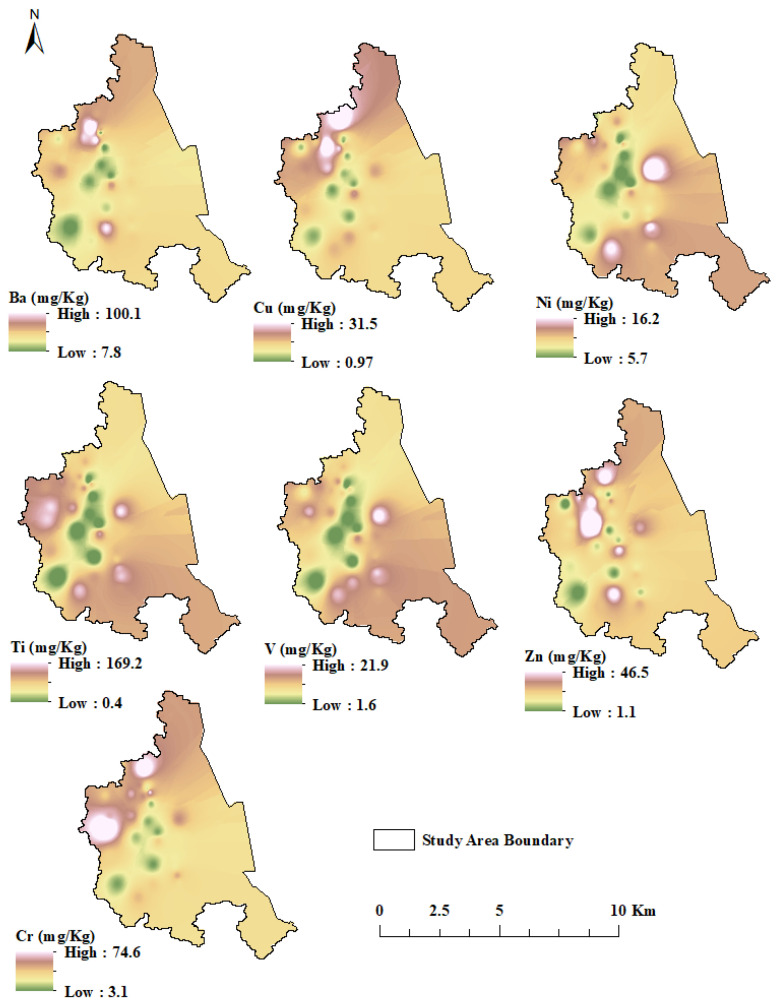
Spatial variability of input–output variables used for modeling.

Basic data statistics were performed using Minitab version 16. Additionally, PCA and CA were equally conducted to differentiate various groups of the analytes with approximate geochemical behaviors and explore the correlations among the various elements. Before the simulation process, the raw data was analyzed and standardized into unit variance as well as zero means. Then, the eleven heavy metals were grouped based on CA. The data used in the modeling process of the current study were taken from our experimental research for trace elements present in the soil in the Dammam region of Saudi Arabia. Three different computational techniques were used in modeling and predicting the Cr concentration (mg/kg) as the dependent (target) variable, whereas the concentrations (mg/kg) of six other trace metals, Ba, Cu, Ni, Ti, V, and Zn, were chosen as the independent (input) variables. The spatial analysis for input–output variables used in this study are presented in Figure 3.

**Figure 4:**
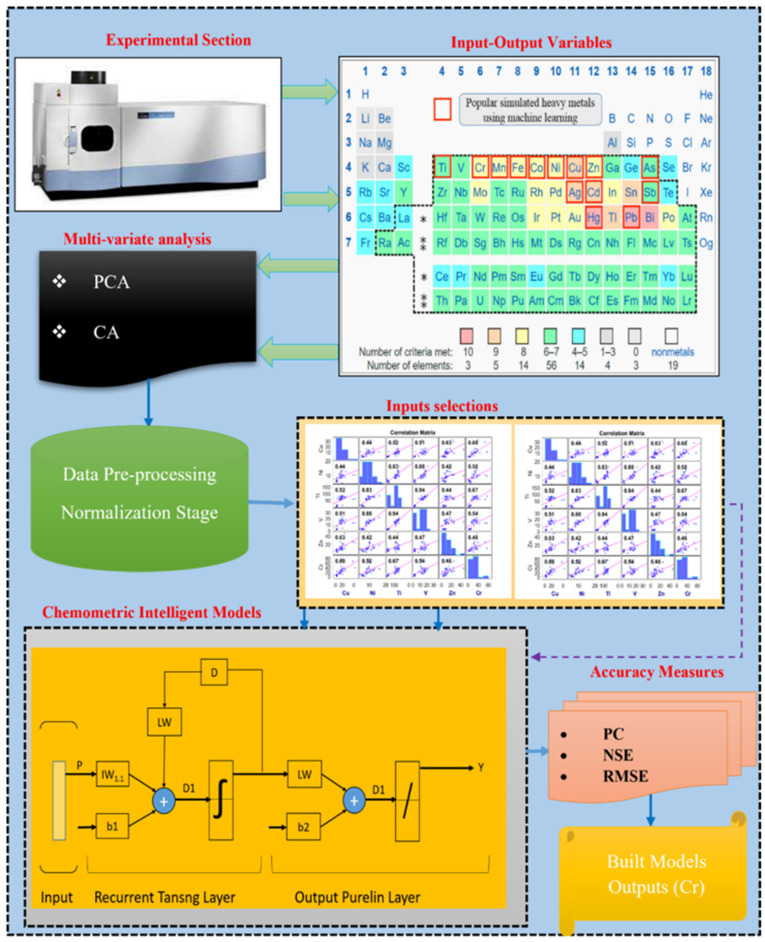
Proposed methodology adopted in this study.

**Figure 5:**
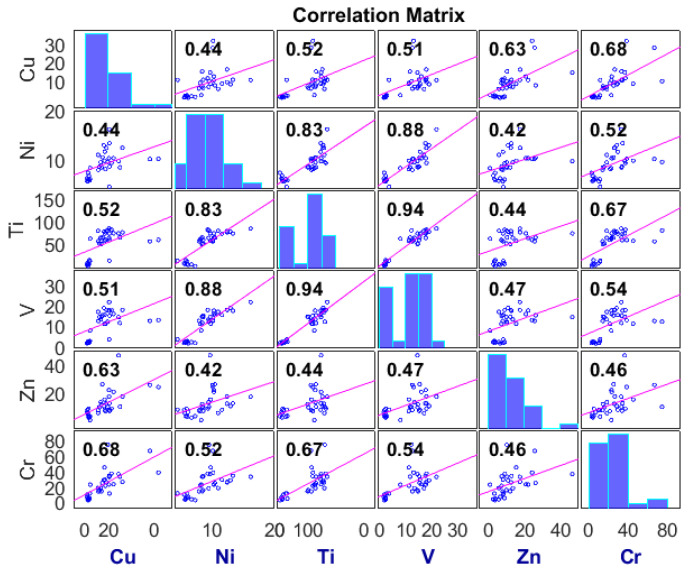
Feature selection of the variables based on the Spearman–Pearson-based correlation technique.

Three different data-driven approaches, including GRNN, Elm NN, and MST, were comparatively used in the current work to determine the best model that can be employed in estimating the concentration of Cr as a potentially toxic trace element to both humans and soil microorganisms. Before starting the estimation step, preliminary data processing techniques were carried out by employing both the AI-based models GRNN and Elm NN, and the linear approach MST (Figure 4). The cross-validation technique was equally utilized to ensure that both under- and overfitting were avoided in both the calibration and validation steps. The data was subsequently divided into 65% calibration and 35% validation. Furthermore, the current study employed the application of the Spearman–Pearson-based correlation technique for the determination of feature selection as well as for understanding the nature of the dataset for the agricultural area (see Figure 5). Based on the correlation performance of the input variables against the target Cr, the independent variables were classified into C1: Zn, Ni, and Ba, and C2: V, Cu, and Ti before the estimation stage.

### 2.5. Generalized Regression Neural Network (GRNN)

**Figure 6:**
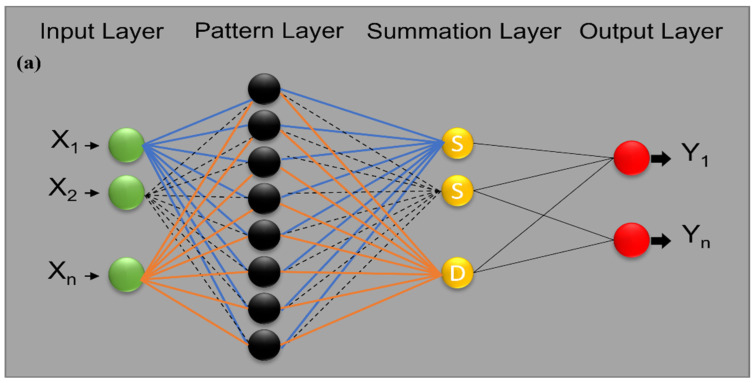

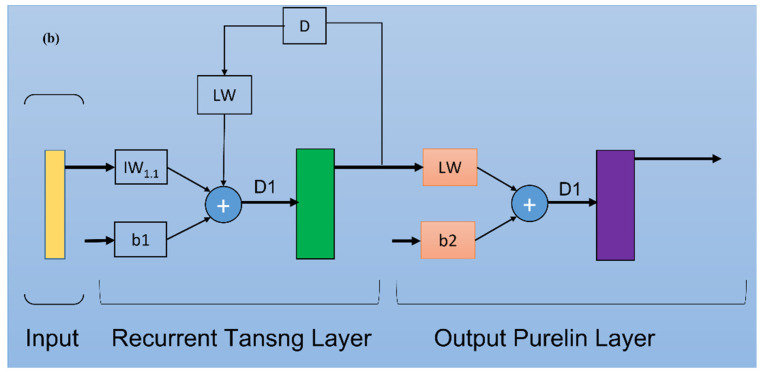
Proposed architecture for (**a**) generalized neural network (GRNN) and (**b**) Elman neural network (Elm NN).

The GRNN was introduced by Specht [26] as the family of ANN models; the models used single-pass learning ANN. The GRNN consists of four layers (input, hidden, summation, output) unlike the typical neural network (see Figure 6a). As a traditional network, all the classifications of neural networks can serve as the multi-input multi-output model; this learning capability also was attributed to GRNN. To understand the learning paradigm of GRNN, the inputs’ player is similar to the classical neural network, where the weight is transposed and the training was associated with a Gaussian kernel’s functions. The major different between GRNN and ANN is that the former is a single-pass network, while the latter uses two passes. This makes the GRNN take less time for training. Secondly, the type of neurons differs for GRNN as well; in GRNN, the weight of each neuron is computed using a special parameter called a smoothing parameter (σ), which is also regarded as the hyper-parameter tuning. The σ is responsible for learning improvements of the models based on the accuracy evaluation [27].

Its general form is depicted below:

(1) $$P_{i}=exp\left(-\frac{{\left(X-X_{i}\right)}^{T}\left(X-X_{i}\right)}{2\sigma^{2}}\right)$$

where X is the input data of the testing dataset, $X_{i}$ is the *i*th input of the training dataset, and *σ* is the smoothing parameter.

### 2.6. Elman Neural Network (Elm NN)

As a subset of ANN, the Elman NN is a recurrent neural network (RNN) that consists of layers, such as those of traditional BPPNNs. The Elm NN has been applied in several fields of science and engineering to solve the problems of simulation, prediction, and estimation [28,29]. Basically, Elm NN consists of four layers, including inputs, hidden, context, and output layers. The interaction between the layers are considered as FFCNs (feed-forward connection networks). The context layers of Elm NN are used to contain the hidden layers’ output values [30]. Elman NN is, generally, regarded as a feedback NN with tap delay layers. As a black-box dynamic model, Elman NN can map both the single- and multiple-input–output relationship; such models can be applied to simplify several derivative calculations, and are highly valued for research purposes. It is evident that a significant amount of research is devoted to showing the advantage of introducing RNNs. System identification has been popular recently, and RNNs such as Elan NN are widely recommended within this field. It is reported that feed-pass loops have the greatest influence on the learning capability of RNNs [31]. In comparison with classical ANNs, RNNs are promising in terms of capturing complex system and complexity behavior (see Figure 6b).

### 2.7. Multivariate Statistical Techniques (MST)

Generally, we have different kinds of multivariate statistical techniques (MST), including multi-linear regression (MLR), stepwise-linear regression (SWLR), interaction-linear regression (ILR), etc. The MST technique is regarded as one of the conventional regression approaches used to understand and model the collinearity of various parameters as targets and independent parameters. Overall, it is described using Equation (4):

(2) $$y=b_{0}+b_{1}x_{1}+b_{2}x_{2}+\ldots b_{i}x_{i}$$

where *y* is the target parameter, $x_{1}$ is the value of the *i*th predictor, $b_{0}$ is the regression constant, and $b_{i}$ is the coefficient of the *i*th predictor.

### 2.8. Evaluation Metrics of the Models

The predictive values were compared with the measured values and then checked using different performance indices to determine various computation methods’ performances. For instance, the current study explores the application of three other statistical indices, namely, the Nash–Sutcliffe coefficient efficiency (*NSE*) and the Pearson coefficient (*PC*), whereby the root mean squared error (*RMSE*) is used in determining the error depicted by each model.

(3) $$NSE=1-\frac{\sum_{j=1}^{N}{\left[{\left(Y\right)}_{obs,j}-{\left(Y\right)}_{com,j}\right]}^{2}}{\sum_{j=1}^{N}{\left[{\left(Y\right)}_{obs,j}-{\bar{\left(Y\right)}}_{obs,j}\right]}^{2}}$$

(4) $$PC=\frac{\sum_{i=1}^{N}\left(Y_{obs}-{\bar{Y}}_{obs}\right)\left(Y_{com}-{\bar{Y}}_{com}\right)}{\sqrt{\sum_{i=1}^{N}{\left(Y_{obs}-{\bar{Y}}_{obs}\right)}^{2}}\sum_{i=1}^{N}{\left(Y_{com}-{\bar{Y}}_{com}\right)}^{2}\;\;\;\;\;\;\;\;\;\;\;}\;\;$$

(5) $$RMSE=\sqrt{\frac{\sum_{i=1}^{N}{\left(Y_{obsi}-Y_{comi}\right)}^{2}}{N}}$$

## 3. Results and Discussion

The American Association of State Highway and Transportation Official (AASHTO) sieve analysis procedure was adopted to assess the concentrations and spatial distributions of different trace elements at AG and ID. This provides a method for addressing soil research through a systematic and thorough methodology. The grain size distribution and soil consistency were used to classify soil characteristics. The topsoil was classified using a unified soil classification method. The geochemical map of the sample sites was created using AutoCAD and ArcMap software. During sample collection, a portable global positioning system (GPS) was used to record sample locations. The geochemical spatial distribution map of each element discovered in the different regions was then created using Surfer 8 software. After analysis, the trace metal content in each place was combined with the geographical coordinates of each sample location obtained during sample collection. The levels of metals are presented below in parentheses, with the mean levels followed by the maximum level detected in (mg/kg) in the sampled locations. Since the Kingdom of Saudi Arabia does not have well-defined guidelines regarding the limits of trace metals in soil, the Canadian Environmental Soil Quality Guidelines (CESQG) standards for the Protection of Environment and Human Health (PEHH) were adopted for comparison purposes.

### 3.1. Spatiotemporal Characteristics of Soil Heavy Metal Contents

Most of the metals followed a general trend in concentration with the highest means found in the sample from the industrial region, then agricultural, and subsequently, the residential locations. The lowest means were found in the background area. The results of the analysis of the topsoil samples are shown in Table 1, along with the allowable limits stated by CESQG for different heavy metals. The concentration of Arsenic (As) was highest in the industrial area (mean = 1.58 mg/kg, maximum = 4.56 mg/kg) followed by the agricultural region (mean = 1.516 mg/kg, max = 3.135 mg/kg), while the lowest was in the residential area (mean = 0.97 mg/kg, max = 2.22 mg/kg). However, none of the samples was tested above the 12 mg/kg threshold. The concentrations of barium (Ba) was highest in the industrial area (mean = 335.5 mg/kg, max = 1966.5 mg/kg), followed by the agricultural (mean = 34.46 mg/kg, max = 100.62 mg/kg) and residential areas (mean = 34.11 mg/kg, max = 98.55 mg/kg). Some of the samples from the industrial areas exceeded the allowable limit of 500 mg/kg. The concentration of cadmium (Cd) was highest in the industrial area (mean = 1.878 mg/kg, max = 28.69 mg/kg), followed by the residential area (mean = 1.878 mg/kg, max = 28.69 mg/kg) and agricultural area (mean = 0.07, max = 1.137). One sample from each industrial and agricultural area measured above the allowable 10 mg/kg limit.

Nevertheless, the amount of Cr was found to be more prevalent in the industrial sample (with mean = 51.77 mg/kg, max = 247.6 mg/kg) than the residential soil samples (mean = 29.64 mg/kg, max = 120.22 mg/kg) and agricultural area (mean = 24.4 mg/kg, max = 74.7 mg/kg). The relative averages of the total samples measured were above the permissible concentration. The concentration of copper (Cu) was highest in the industrial area (mean = 11.0 mg/kg, max = 95.75 mg/kg), followed by the agricultural (mean = 8.74, max = 31.64) and residential areas (mean = 4.38 mg/kg, max = 19.17 mg/kg). Two of the samples from the industrial area exceeded the allowable limit of 63 mg/kg. The concentration of mercury (Hg) was highest in the industrial area (mean = 0.11 mg/kg, max = 1.44 mg/kg), followed by the agricultural (mean = 0.087 mg/kg, max = 1.02 mg/kg) and residential areas (mean = 0.06 mg/kg, max = 0.59 mg/kg). None of the samples exceeded the allowable limit of 6.6 mg/kg. The concentration of nickel (Ni) was the highest in the industrial area (mean = 13.14 mg/kg, max = 45.2 mg/kg), followed by the agricultural (mean = 9.22 mg/kg, max = 16.25 mg/kg) and residential areas (mean = 6.44 mg/kg, max = 13.23 mg/kg). None of the samples exceeded the allowable limit of 50 mg/kg. The concentration of lead (Pb) was highest in the industrial area (mean = 11.42 mg/kg, max = 100.25 mg/kg), followed by the agricultural (mean = 6.49 mg/kg, max = 52.35 mg/kg) and residential areas (mean = 4.79 mg/kg, max = 25.6 mg/kg). None of the samples exceeded the allowable limit of 140 mg/kg. Similarly, the concentration of vanadium (V) was highest in the industrial area (mean = 13.11 mg/kg, max = 20.42 mg/kg), followed by the agricultural (mean = 11.52 mg/kg, max = 21.89 mg/kg) and residential areas (mean = 7.0 mg/kg, max = 17.73 mg/kg). None of the samples exceeded the allowable limit of 130 mg/kg. The concentration of zinc (Zn) was highest in the industrial area (mean = 65.44 mg/kg, max = 676.5 mg/kg), followed by the agricultural (mean = 12.43 mg/kg, max = 46.25 mg/kg) and residential areas (mean = 8.47 mg/kg, max = 39.39 mg/kg). None of the samples exceeded the allowable limit of 200 mg/kg.

### 3.2. Results of Multivariate Statistics

For the agricultural area, cluster analysis (CA) was performed to distinguish different groups of elements with approximate geochemical behaviors. The results reveal that, based on the close relations between heavy metals in the soil, As, Cd, Hg, Cu, V, and Pb could be sorted into a single group; Ni, Zn, Cr, and Ba in another group, and Ti as the third group To further explore the relationships among the eleven elements, PCA was performed. The PCA showed that ~74.4% of the data variance could be explained by the first three principal components (factors). These three principal components were extracted with an eigenvalue >1. In particular, the eigenvalues of component 1 (~44% of inertia), component 2 (16.8% of inertia), and component 3 (13.6% of inertia) were 4.8, 1.8, and 1.5, respectively. Component 1 was mainly related to V, Cr, and Cu, component 2 was associated mainly with Pb and hg, and component 3 was related to As.

In addition, the CA analysis for the industrial area reveals that As, Hg, Cu, and Pb could be sorted into a single group; V, Ni, Cr, Cd, and Zn in another group, and Ti and Ba as the third group Furthermore, the PCA analysis shows that ~77% of the data variance can be explained by the first three principal components (factors). Ni, Cr, and Cu are the predominant elements in the first component, and Cd, V, and Ti are the predominant elements in the second component, while the contribution to the third component was mainly due to As and Hg. These components exhibit eigenvalues more significant than one and have therefore been considered here. In particular, the eigenvalues of component 1 (~48.9% of inertia), component 2 (17.4% of inertia), and component 3 (10.7% of inertia), were 5.38, and 1.91, and 1.18, respectively.

The CA analysis for the residential area reveals that As, Pb, Cu, V, Hg, and Zn could be sorted into a single group; Ni, Cd, Ba, and Cr in another group, and Ti as the third group. Moreover, PCA analysis reflects that ~78% of the data variance can be explained by the first their principal components with the eigenvalues of component 1 (~52.7% of inertia), component 2 (15.4% of inertia), and component 3 (9.9% of inactivity) as 5.79, 1.69, and 1.08, respectively. V, Ti, Ni, and Ba are the predominant elements in the first component, Hg and Cr are the predominant elements in the second component, while the contribution to the third component was mainly due to As, Hg, and Pb. However, CA analysis for the background area reveals that As, Hg, Cu, Pb, Ni, V, and Zn could be sorted into one group; Ba, Cr, and Cd in another group, and Ti as the third group. PCA for the background area shows that ~84.1% of the data variance can be explained by the first two principal components (factors). Particularly, the eigenvalues of component 1 (~52.7% of inertia) and component 2 (15.4% of inertia) were 5.8 and 1.7, respectively. V, Cu, Ti, and Ni were the predominant elements in the first component, while the contribution to the second component was mainly due to As, Hg, and Cr.

### 3.3. Results for AI-Based Computational Models

Traditional and classical trivial regression approaches have been utilized for the extraction, analytical exploration, and estimation of trace metals, despite their numerous issues and limitations. Based on this, the AI-based technique was developed to enhance and improve the performance prediction of trace elements from the soil, water, and air. One of the primary motivations of this article is the implementation of two different AI techniques based on the most widely employed neural networks (NN). Different training networks inform GRNN, and Elm NN models integrated with the MST linear approach to predict Cr concentration (mg/kg) in the AG regions of Dammam, Saudi Arabia. The proper selection of hyper tuning parameters plays a crucial role in the modeling process and model accuracy. Therefore, the tuning parameters used for NN, for example, are 1000, 0.01, and 0.0001 for the maximum number of iterations, learning rate, and MSE, respectively. For the development of NN, formulating a proper number of hidden nodes is the most important aspect, as such hidden layers were identified using (2n1/2 + m) to (2n + 1), where n is the number of input neurons and m is the number of output nodes. The optimal structure was determined using the range of 2–10 hidden nodes, and 15–60 calibration epochs. The major advantage of GRNN over other types of neural networks is that there is only a single hyper-parameter, namely, the sigma. The random search strategy is to find a close-to-optimal value of the sigma by using various random numbers. Elman NN has been designed and trained with a conventional learning algorithm, with the values obtained in the initial iteration of the learning process as the initial set values for optimal tuning algorithms.

In the validation process, different types of validation approaches can be applied, including cross-validation, which is called k-fold cross-validation; others are holdout, leave one out, and so on. We applied k-fold cross-validation in this study. The sensitivity and importance of the sampling sites and the nature of the dataset were currently considered of global attention. Based on Yassen, [32], Cr is considered one of the most utilized heavy metals using different computational approaches due to its hazardous impact on both humans and the environment. Moreover, the AI-based technique as a cost-effective approach provides economical and efficient benefits to environmental policymakers, especially those dealing with trace metals. The performance of the two classes (C1 and C2) of the utilized models in the current study (GRNN, Elm NN, and MST) was determined using three different performance metrics, NSE, PC, and RMSE, as mentioned in the previous section.

The predicted outcomes of the models in both the calibration and validation steps are shown in Table 2. The calibration step and C2 class showed superior performance to the validation step and C1 class, respectively. The comparative performance of the models (GRNN, Elm NN, and MST) showed different predictability of the Cr concentration (mg/kg) due to the reactive nature of each model network towards the learning process the dataset. Furthermore, this indicates the need to utilize various metrics and visualizations for comparative analysis of the models’ performance. The quantitative interpretation of the models, as indicated in Table 2, shows the robustness of the AI-based models (GRNN and Elm NN) over the classical MST model, even though only the GRNN showed a performance with a minimum or higher NSE value of 0.8 in both the two input combinations (C1 and C2) and two steps (calibration and verification), respectively.

**Figure 7:**
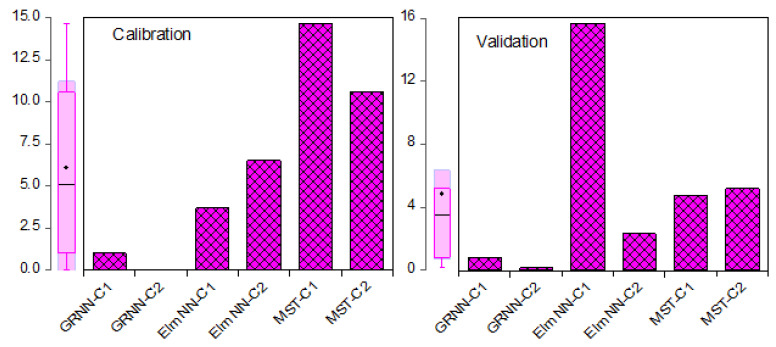
Comparative RMSE performance of the GRNN, Elm NN, and MST techniques for the Cr concentration (mg/kg) estimation in both calibration and validation phases.

The Elm NN-C2 showed higher performance than Elm NN-C1 in the calibration and validation steps. At the same time, both MST-C1 and MST-C2 failed to estimate the Cr concentration (mg/kg) in both the calibration and validation phases with significant performance. Moreover, the comparative performance of the models indicated in Table 2 depicts that GRNN as an AI-based model can boost the performance of the classical linear MST model up to 27% and 94.6% in the calibration and validation phases, respectively, for the C2 input combination. The performance of the models can be better pictured using different visualizations to ease grasping of the performance results of the models. For instance, Figure 7 indicates the comparative error performance of the models based on their respective RMSE values in both the calibration and validation stages.

**Figure 8:**
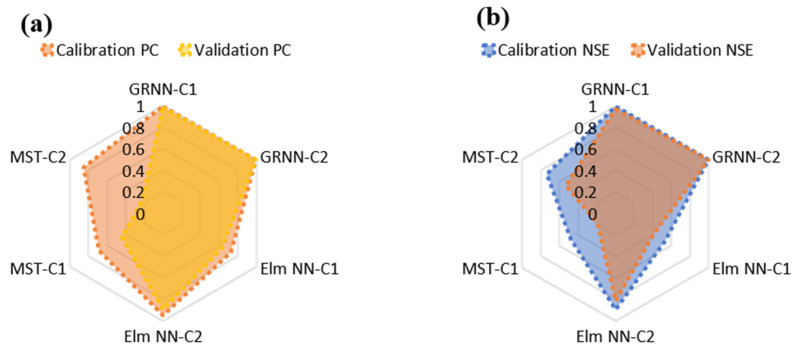
Comparative (**a**) PC and (**b**) NSE performance of the GRNN, Elm NN, and MST techniques for the Cr concentration (mg/kg) estimation in both calibration and validation.

**Figure 9:**
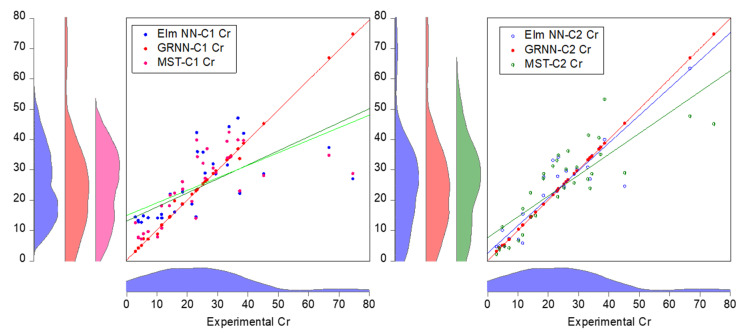
Scatter plots of the GPRNN, Elm NN, and MST for both C1 and C2.

Moreover, the comparative performance of the models based on their goodness-of-fit in the form of PC and NSE can be visualized using the radar plot as shown in Figure 8. To better understand the data-driven techniques’ performance of each of the input combinations (C1 and C2), a visualization based on graphical illustration using time series and scatter plots was performed. These graphs can be utilized in evaluating the precision of the developed models. Based on the goodness-of-fit-values, the scatter plots illustration of the models is shown in Figure 9.

**Figure 10:**
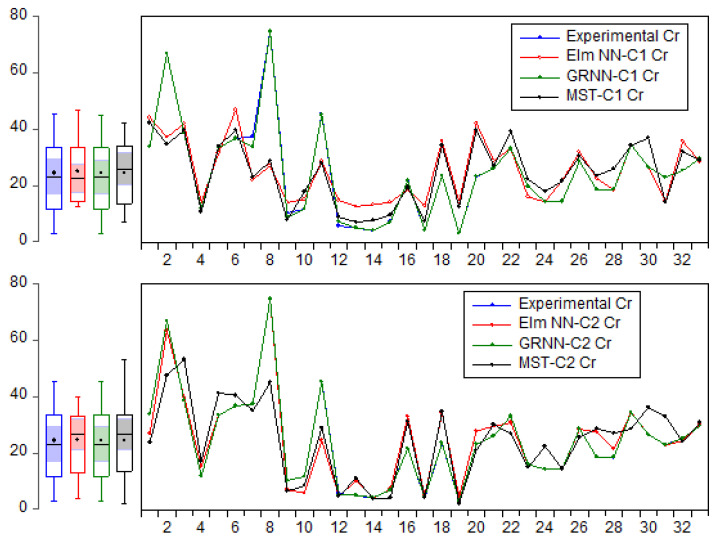
Trend of estimation of the GRNN, Elm NN, and MST models against the experimental Cr concentration (mg/kg).

The scatter plot is one of the effective visualizations used in evaluating the predictability performance of various data-driven approaches, indicating the degree of deviation of the predicted values against the experimental values. Moreover, as shown in Figure 10, the time series depicts the trend of estimation of each data-driven approach (GRNN, Elm NN, and MST models) against the experimental Cr concentration (mg/kg). Based on the numerical and visualized performance of the models used in the current study for the estimation of Cr concentration (mg/kg), the models can be arranged based on their performance skills as follows: GRNN-C2 > GRNN-C1 > Elm NN-C2 > Elm NN-C1 > MST-C2 > MST-C1.

### 3.4. Discussion of Spatiotemporal Analysis

An analysis of topsoil can be used to estimate trace metal pollution in our environment [33]. Therefore, regular assessment of metal contamination in topsoil is necessary [34]. The USA’s standards for maximum contaminant levels are even higher than the Canadian allowable limits. The abundance of As in agricultural areas can be attributed to insecticides such as hydrogen arsenate on fruit trees [35]. Another valid reason for high levels of As in agricultural areas can also be attributed to the use of arsenic as a food additive in poultry to boost their weight, enhance their feeding, and preclude diseases [36], or the use of Ba titanate in a promising electro-ceramic procedure [37] and the use of mineral barite as an additive in oil well-drilling muds.

Further analysis indicated that the result of Cd is within the standard allowable limit for the three sample locations (background, residential, industrial). It is indicated in the industrial and residential zones that one sample has exceeded the allowable limit. This could be due to several industrial activities in the study locations. However, it can also be seen that some deposited material forms the illegal sources and other sources contained Cd. For Cr, the elevated levels might be associated with both natural, manmade, and atmospheric deposition of compound containing Cr. Samples 3, 5, 18, and 24 displayed high levels of Cr in the residential soil samples due to accidental release, wood additives, or atmospheric deposition of compounds. The Cr compound was also found to be at elevated levels for samples 13, 14, 15, 17, 18, and 30, which is due to the industrial application of Cr such as in the paint industry, tanning, roofing, dyes, etc. [38].

**Figure 11:**
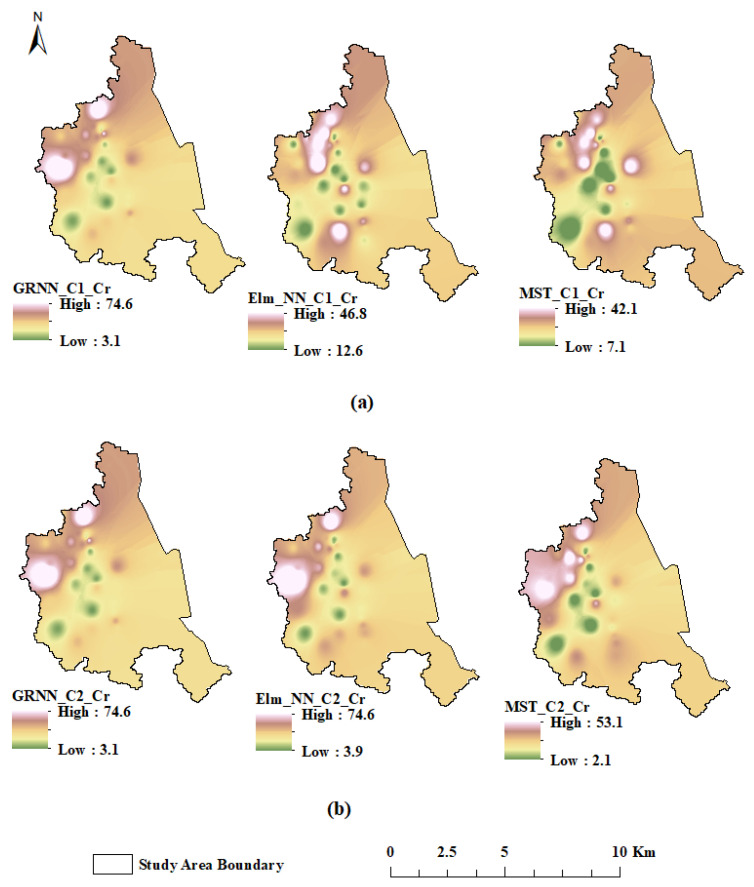
Predicted spatial analysis for both (**a**) C1 and (**b**) C2 combination.

With regards to Cu compounds, the increasing levels in 2 out of 33 industries were linked to the substantial production of electrical cables, plumbing indifferent industries across the study area [39]. Other factors could also be attributed to atmospheric deposition or accidents containing Cu compounds. Meanwhile, in order to reduce human exposure to Cu compounds, extreme care needs to be exercised when working with Cu compounds [40]. The adopted guidelines for the level of Zn in the soil specify 200 mg/kg as the allowable limit, and all the analyzed samples fell within this range. Thus, no immediate threat is in sight from the tested trace metals in the studied areas of Dammam. However, proper monitoring is essential to keep these levels of metals in check. Rules must also be implemented to transport and handle chemicals that contain these trace metals. The predicted spatial analysis results using AI-based models are presented in Figure 11.

## 4. Conclusions and Recommendations

Research on the soil environment is necessary for sustainable development and improvement of the quality of life. As mentioned in the previous sections, there is very little information available regarding toxic metal contamination of topsoil in the Dammam area. So, the main motivation for this study was to assess the topsoil in the area to determine trace metal concentration, their “hot spot” (area of high concentrations of trace metals), and spatial distribution. These will help in assessing the health conditions of the topsoil in the region and may later be used for setting up remediation measures or land use allocations. This study outcome may also create further research in environmental assessment and monitoring. The current study analyzed various soil samples from different locations in the Dammam region of the Saudi Area, which equally determined the concentration of ten other heavy metals. The attention to these heavy metals almost follows the same trend, whereby they were all found at relatively higher amounts in the study locations. Most of the concentrations of the heavy metals were found to be within the accepted range, but some were found to exceed the accepted range. Conversely, based on their average concentrations, none of the heavy metals exceed their allowable threshold in the whole experiment. These findings, at first instance, provide a relief that the concentration of these heavy metals determined was found to be within the allowable range. Hence, there is no alarming threat to human life and the ecosystem.

Most of the metals followed a general trend in concentration with the highest means found in the sample from the industrial region, then agricultural, and subsequently, the residential locations. The lowest means were found in the background area. The amount of Cr was found to be more prevalent in the industrial samples than in the residential soil samples and agricultural areas. The relative average of the total samples measured was above the permissible concentration. The concentration of copper (Cu) was highest in the industrial area, followed by the agricultural and residential areas. Nevertheless, higher concentrations of these metals compared to the control and residential areas suggest careless handling of chemicals in these areas. It is, therefore, evident that if proper monitoring schemes are not devised to plug any existing loopholes, it can lead to greater exposure of these metals into the ecosystem. Eventually, various aquatic life, animals, and plants might be at risk, which is related to heavy metals exposure. Furthermore, the prediction approach showed the performance abilities of the AI-based models (GRNN and Elm NN) over the classical linear MST approach in predicting the concentration (mg/kg) of Cr trace elements. However, the results based on the numerical and visualized performance obtained at a particular stage indicate the need for employing more robust techniques such as the metaheuristic approaches and ensemble machine learning to improve the performance prediction. The research also indicated the smaller number of the data instances as the limitation of the research as the machine learning needs a huge amount of data in order to represent the real information of the scenario.

## Figures and Tables

**Table 1:** Concentration of trace metals in the Dammam area.

Agriculture Area										
Element	As	Ba	Cd	Cr	Cu	Hg	Ni	Pb	V	Zn
Unit	mg/kg	mg/kg	mg/kg	mg/kg	mg/kg	mg/kg	mg/kg	mg/kg	mg/kg	mg/kg
Allowable limit	**12**	**500**	**10**	**64**	**63**	**6.6**	**50**	**140**	**130**	**200**
MCLs	12	750	1.4	64	63	6.6	50	70	130	200
Min	0.45	7.8	0	3.03	0.97	0	4.6	0.9	1.635	1.015
Max	3.135	100.62	1.135	74.7 *	31.64	1.025	16.25	52.35	21.885	46.25
Mean	1.516	34.46	0.07	24.4	8.74	0.087	9.22	6.49	11.52	12.43
No. of samples	33	33	33	33	33	33	33	33	33	33
Industrial Area										
Element	As	Ba	Cd	Cr	Cu	Hg	Ni	Pb	V	Zn
Unit	mg/kg	mg/kg	mg/kg	mg/kg	mg/kg	mg/kg	mg/kg	mg/kg	mg/kg	mg/kg
Allowable limit	**12**	**500**	**10**	**64**	**63**	**6.6**	**50**	**140**	**130**	**200**
MCLs	12	2000	22	87	91	50	50	600	130	360
Min	0.52	0.0	0	0.12	0.18	0.01	4.76	0.04	0.09	0.1
Max	4.56	1966.5 *	28.69 *	247.6	95.75 *	1.44	45.2	100.25	20.42	676.5 *
Mean	1.58	335.35	1.878	51.77	11.0	0.11	13.14	11.42	13.11	65.44
No. of samples	33	33	33	33	33	33	33	33	33	33
Residential Area										
Element	As	Ba	Cd	Cr	Cu	Hg	Ni	Pb	V	Zn
Unit	mg/kg	mg/kg	mg/kg	mg/kg	mg/kg	mg/kg	mg/kg	mg/kg	mg/kg	mg/kg
Allowable limit	**12**	**500**	**10**	**64**	**63**	**6.6**	**50**	**140**	**130**	**200**
MCLs	12	500	10	64	63	6.6	50	140	130	200
Min	0.13	0.33	0	0.07	0.04	0.0	2.25	0.08	0.02	0.01
Max	2.22	98.55	23.01	120.2	19.17	0.59	13.23	25.6	17.73	39.39
Mean	0.97	34.11	1.87	29.64	4.38	0.06	6.44	4.79	7.00	8.47
No. of samples	33	33	33	33	33	33	33	33	33	33

* The value that exceeds the permissible concentration.

**Table 2:** Performance of the GRNN, Elm NN, and MST techniques.

	Calibration Phase		
	NSE	PC	RMSE
GRNN-C1	0.998	0.999	0.990
GRNN-C2	0.999	0.999	0.027
Elm NN-C1	0.524	0.723	3.677
Elm NN-C2	0.898	0.948	6.503
MST-C1	0.481	0.693	14.688
MST-C2	0.731	0.855	10.575
	**Validation Phase**		
GRNN-C1	0.976	0.988	0.829
GRNN-C2	0.999	0.999	0.158
Elm NN-C1	0.407	0.638	15.689
Elm NN-C2	0.812	0.901	2.315
MST-C1	0.194	0.441	4.785
MST-C2	0.53	0.230	5.189

## Data Availability

Data available upon request.

## Sample Availability

Samples of the compounds are available from the authors.
